# Autism: Where Genetics Meets the Immune System

**DOI:** 10.1155/2012/486359

**Published:** 2012-09-05

**Authors:** Antonio M. Persico, Judy Van de Water, Carlos A. Pardo

**Affiliations:** ^1^Child and Adolescent Neuropsychiatry Unit, University Campus Bio-Medico, Via Alvaro del Portillo 21, 00128 Rome, Italy; ^2^Laboratory of Molecular Psychiatry and Neurogenetics, University Campus Bio-Medico, Via Alvaro del Portillo 21, 00128 Rome, Italy; ^3^Division of Rheumatology/Allergy and Clinical Immunology, UC Davis, 451 Health Science Drive, Suite 6510, GBSF, Davis, CA 95616, USA; ^4^Department of Neurology, Johns Hopkins University School of Medicine, 600 N. Wolfe Street, Baltimore, MD 21287, USA

Individuals with autism often display immune abnormalities in the form of altered cytokine profiles, autoantibodies, and changes in immune cell function. Are they mere “smoke” or real “fire”? Given what we now know about the cross-talk between the immune and nervous systems, one must ask: are these immune abnormalities simply bystander effects of genetic/genomic variants directly responsible for abnormal neurodevelopment, or is there a sizable subgroup of patients with autism spectrum disorder (ASD), in which a dysfunctional immune system plays a critical mediator role in the pathogenic chain of events leading to the disorder? Is autism one of many genetic/genomic conditions, Down syndrome representing the best-known example, accompanied by immune abnormalities not primarily responsible for changing the trajectory of neurodevelopment? Or could conceivably an unfortunate combination of genetic/genomic susceptibility and environmental factors converging onto the same individual affect neurodevelopment through immune-mediated mechanisms, such as fetomaternal incompatibility, autoimmunity, abnormal neuroinflammation, and so on?

This special issue contains six contributions aimed at addressing different aspects of this question.

The paper by N. Momeni et al. documents significantly elevated plasma levels of factor I among ASD children compared to controls. Factor I is a plasma enzyme responsible for degrading complement factor 3b, which in turn is the major opsonin in the complement system enabling phagocytosis of microbial agents. Higher factor I levels can be interpreted as either primary (i.e., conferring vulnerability toward microbial infections) or as a secondary event part of a broader immunomodulatory response aiming to blunt an inflammatory process. F1 plasma levels suggest that this process could play a more relevant role in males and in small ASD children.

The contribution by S. Rose et al. describes reduced glutathione-mediated redox/antioxidant capacity both inside primary leukocytes and in the plasma of ASD children. Consistently with this intracellular and extracellular imbalance of the glutathione redox status, intracellular production of free radicals is also enhanced, especially in 30% of ASD cases. This and several previous studies provide converging evidence of excessive oxidative stress likely leading to abnormal mitochondrial function in a similar percentage of autistic individuals.

Along similar lines, M. I. Waly et al. direct their attention on the complex array of pathophysiological consequences produced by enhanced oxidative stress. In addition to imbalanced glutathione redox status, reduced methionine synthase activity is particularly interesting, as it results in lower availability of S-adenosyl-methionine and consequently blunted DNA methylation. Curiously, a similar abnormality underlies autistic features also in two entirely different contexts, namely, children exposed prenatally to valproic acid and in Rett syndrome girls, carrying MECP2 mutations.

The review by S. D. Bilbo et al. presents a stimulating hypothesis, linking ASD to inflammatory, allergic, and autoimmune diseases. These “hyperimmune” disorders are known to share steeply increasing incidence rates over the last decades, as well as significant loading in many families with children with autism. In the authors' view, they also share an inappropriate activation of the immune system due to biome depletion in postindustrial societies, possibly suggesting preventive strategies based on biome reconstitution.

The paper by C. Onore et al. reports an impressive threefold decrease in EGF plasma levels among small ASD children compared to controls. EGF is involved in wound healing in the skin, in the gastrointestinal, and respiratory systems, as well as in the central nervous system where this neurotrophic factor supports both adult subventricular zone and midbrain dopaminergic neurons, in addition to stem cell proliferation.

A. R. Torres et al. review immune abnormalities in autism, focusing on HLA gene roles as potential contributors to autism pathogenesis through several distinct mechanisms. HLA genes or haplotypes have been found associated with several autoimmune diseases, but strong associations with autism have also been reported.

These contributions, while not providing the ultimate answers, do add some new pieces to the autism puzzle, providing further support to the idea that immune abnormalities may play a pathophysiologically relevant role in a subgroup of families with autistic children. Future studies of the interaction of the immune and central nervous system are needed to clarify in more detail how neuroimmune mechanisms influence the enhancement of the neurodevelopmental disarray and disturbances of neurobehavioral trajectories determined by genetic and environmental factors. The heuristic potential of this line of investigation, both in terms of prevention and clinical therapeutics, ensures continued efforts until a more definitive answer can be given to our initial question, so crucial in today's autism field.

*Antonio M. Persico* *Judy Van de Water* *Carlos A. Pardo*

